# Transperineal total mesorectal excision for rectal cancer on the residual rectum after multiple abdominal surgeries in a patient with Crohn’s disease: a case report

**DOI:** 10.1186/s40792-021-01206-7

**Published:** 2021-05-13

**Authors:** Shin Emoto, Shigenori Homma, Tadashi Yoshida, Nobuki Ichikawa, Yoichi Miyaoka, Hiroki Matsui, Ryo Takahashi, Keita Ishido, Takuya Otsuka, Tomoko Mitsuhashi, Takehiko Katsurada, Akinobu Taketomi

**Affiliations:** 1 Department of Gastroenterological Surgery I, Hokkaido University Graduate School of Medicine, Kita 15 Nishi 7, Kita-ku, Sapporo, Hokkaido 060-8638 Japan; 2 Department of Surgical Pathology, Hokkaido University Hospital, Kita 14 Nishi 5, Kita-ku, Sapporo, Hokkaido 060-8648 Japan; 3 Department of Gastroenterology and Hepatology, Hokkaido University Faculty of Medicine and Graduate School of Medicine, Kita 15 Nishi 7, Kita-ku, Sapporo, Hokkaido 060-8638 Japan

**Keywords:** Rectal cancer, Crohn’s disease, History of surgery, Laparoscopic surgery, Transperineal total mesorectal excision

## Abstract

**Background:**

The improved prognosis of Crohn’s disease may increase the opportunities of surgical treatment for patients with Crohn’s disease and the risk of development of colorectal cancer. We herein describe a patient with Crohn’s disease and a history of multiple surgeries who developed rectal stump carcinoma that was treated laparoscopically and transperineally.

**Case presentation:**

A 51-year-old man had been diagnosed with Crohn’s disease 35 years earlier and had undergone several operations for treatment of Crohn’s colitis. Colonoscopic examination was performed and revealed rectal cancer at the residual rectum. The patient was then referred to our department. The tumor was diagnosed as clinical T2N0M0, Stage I. We treated the tumor by combination of laparoscopic surgery and concomitant transperineal resection of the rectum. While the intra-abdominal adhesion was dissected laparoscopically, rectal dissection in the correct plane progressed by the transperineal approach. The rectal cancer was resected without involvement of the resection margin. The duration of the operation was 3 h 48 min, the blood loss volume was 50 mL, and no intraoperative complications occurred. The pathological diagnosis of the tumor was type 5 well- and moderately differentiated adenocarcinoma, pT2N0, Stage I. No recurrence was evident 3 months after the operation, and no adjuvant chemotherapy was performed.

**Conclusion:**

The transperineal approach might be useful in patients with Crohn’s disease who develop rectal cancer after multiple abdominal surgeries.

## Background

As the treatment of Crohn’s disease (CD) has progressed, its prognosis has improved. Accordingly, the opportunity for patients with CD to undergo surgical treatment has increased. However, the longer duration of Crohn’s colitis may increase the risk of cancer [1]. The relative risk of developing colorectal cancer is reportedly 13 times higher in patients with the colonic type of CD than in the general population [2]. Moreover, cancerization at the residual rectum or rectal stump after total colectomy may become a concern in patients with colonic CD [3, 4]. Surgery for rectal stump carcinoma may be technically demanding because of patients’ history of multiple surgeries.

Local recurrence is a potential complication after surgery for low rectal cancer. Complete total mesorectal excision (TME) with a negative circumferential resection margin (CRM) is important to prevent local recurrence. However, achieving TME with a negative CRM is difficult in patients with obesity, a contracted pelvis, or a bulky tumor [5, 6]. Transanal TME (Ta-TME) is considered to resolve these difficulties. Ta-TME is a technique known as the “bottom up” procedure of TME. This approach provides clear visualization of the dissection plane from the anus or low rectum, which may facilitate improvement of TME quality and reduction of a positive CRM and postoperative complications [7, 8]. Ta-TME is also reportedly useful for patients with a history of multiple abdominal surgeries [9], which causes abdominal adhesion and makes the abdominal approach difficult. Especially, the approach from the bottom was called as “transperineal approach” when the anus was removed [10].

In this report, we describe a patient with CD who developed rectal cancer at the residual rectum after multiple abdominal surgeries. The rectal cancer was successfully treated with transperineal(Tp)-TME.

## Case presentation

The patient was a 51-year-old man with a history of renal failure due to acute rapidly progressive glomerulonephritis that had been diagnosed 2 years earlier and required treatment with artificial hemodialysis. He had been diagnosed with CD 35 years earlier and underwent ileocecal resection. He developed a CD-induced vesicoileal fistula and vesicosigmoid fistula and underwent right colectomy, sigmoid colectomy, closure of the bladder fistula, small bowel resection, and ileostomy 23 years earlier. After this operation, the estimated length of the remaining small bowel was 1 m. Since then, he had received home parenteral nutrition because of short bowel syndrome. He also underwent total colectomy 13 years earlier because he had developed extensive colitis. He currently desired renal transplantation for chronic renal failure and was examined in our hospital. Pre-transplantation colonoscopic examination led to a diagnosis of rectal cancer of the remaining rectum. He was then referred to our department.

**Fig. 1:**
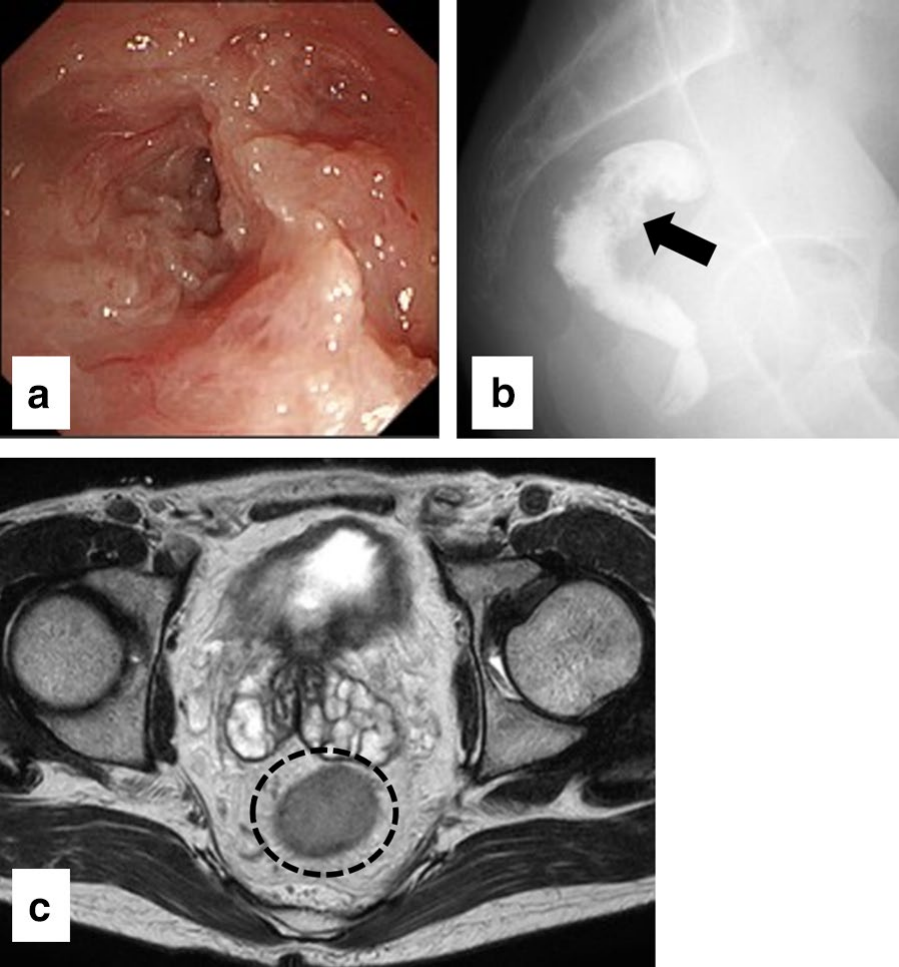
Preoperative examination findings. **a** Colonoscopy showed a circumferential tumor in the residual rectum. **b** Contrast enema showed the tumor located at the low rectum (black arrow). **c** Magnetic resonance imaging revealed the circumferential tumor at the low rectum (dashed circle). The depth of wall invasion by the tumor was estimated as T2 because the muscle layer of the rectum was continuous

On physical examination, his height was 150.7 cm, weight was 38.1 kg (dry weight), and body mass index was 16.8 kg/m^2^. A single ileostomy at the right lower abdomen and a scar from a midline incision extending from the epigastric to suprapubic region were observed. Digital examination could not be performed because of stenosis of the anus secondary to the previous anal fistula, which had cured and showed no evidence of canceration. On hematological examination, his blood urea nitrogen concentration was 43 mg/dL, creatinine concentration was 12.16 mg/dL (before hemodialysis), carcinoembryonic antigen concentration was 1.8 ng/mL, and CA19-9 concentration was 24.4 ng/mL. Colonoscopy showed a circumferential tumor at the remaining rectum (Fig. 1a), and biopsy revealed well-differentiated adenocarcinoma. A contrast enema revealed a 6-cm defect at the remaining rectum around the pritoneal reflection (Fig. 1b). Computed tomography examination revealed wall thickening of the remaining rectum but no swollen regional lymph nodes or distant metastasis. Magnetic resonance imaging showed that the estimated depth of wall invasion by the tumor was T2 because the muscle layer was continuous (Fig. 1c). The preoperative diagnosis of the rectal cancer was clinical T2N0M0, Stage I.

**Fig. 2:**
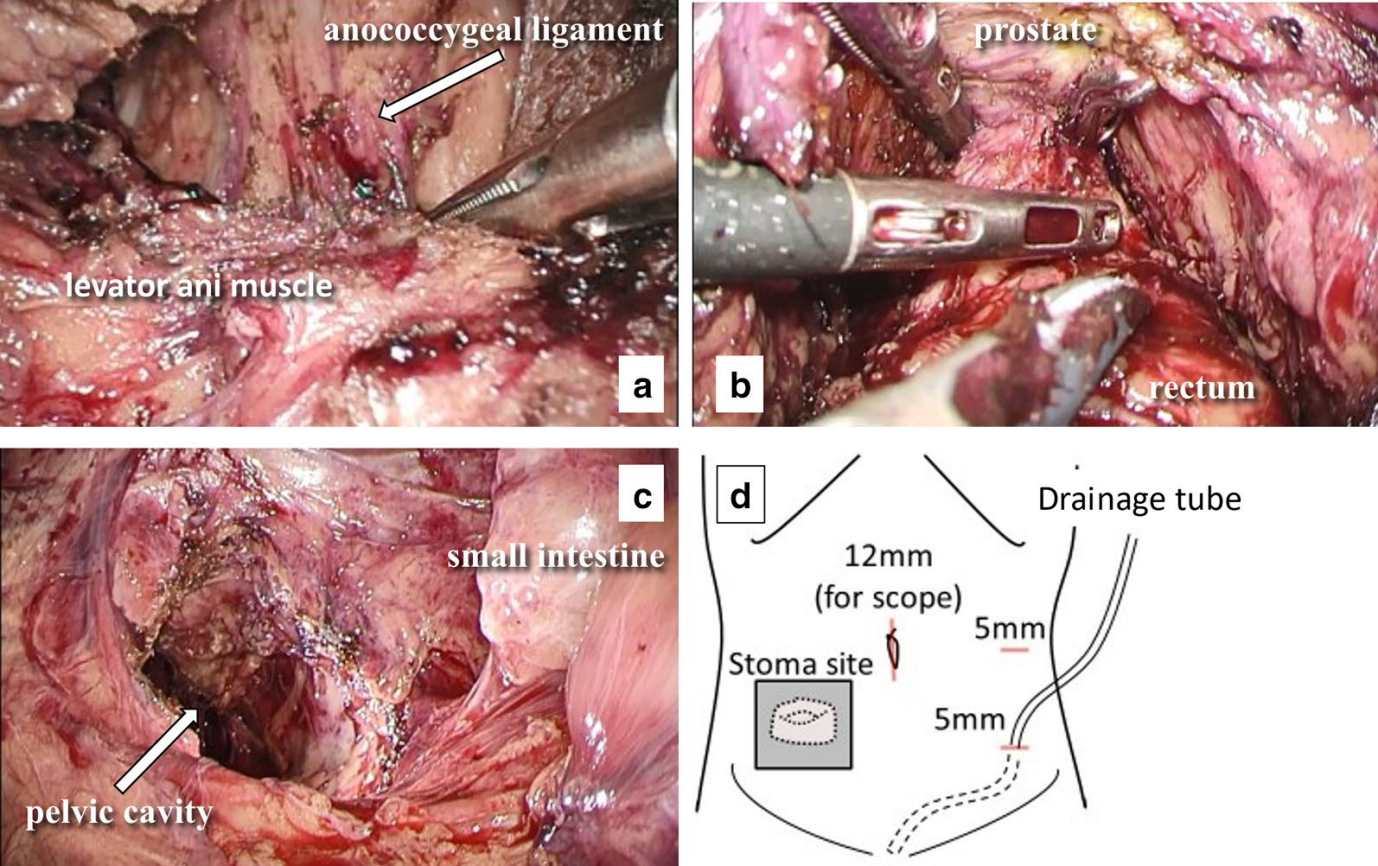
Operative views. **a** Transanal view. After dissecting the levator ani muscle, the anococcygeal ligament was explored. **b** Transabdominal (laparoscopic) view. After rendezvous of the transanal and transabdominal space, the residual rectum was dissected from the dorsal side to lateral side. The prostate was explored laparoscopically, and the layer between the prostate and the residual rectum was dissected with support from the transanal approach to avoid injuring the rectourethral muscle and the urethra. **c** Transabdominal (laparoscopic) view after resection of the residual rectum. Strong adhesion of the small intestine to the pelvic wall was observed, and the natural pelvic anatomy was unclear because of the history of multiple surgeries. **d** Schema of the intraoperative overview of the abdomen. During the operation, the end ileostomy was covered by gauze and plastic film. A drainage tube was inserted from the left lower incision, through which a port was inserted, to the pelvic cavity after the operation

Severe intra-abdominal adhesions were expected because of the patient’s history of multiple surgeries; therefore, we performed an operation with both laparoscopic transabdominal and transperineal approaches. The transabdominal procedure and transperineal approach were carried out concomitantly. For the transabdominal approach, three ports were placed in the abdomen. One of the ports was placed in the umbilicus for scope insertion, and the other two were placed on the left side of abdomen for the laparoscopic operator. The transperineal approach was concurrently implemented. First, two purse-string sutures were placed to close the rectal lumen and prevent cancer cell dissemination. The scar due to the previous anal fistula existed, but could be easily dissected. After dissecting the perianal skin and perirectal soft tissue, a GelPOINT Path transanal access platform (Applied Medical, Rancho Santa Margarita, CA, USA) was inserted and an AirSeal System (CONMED, Utica, NY, USA) was used for carbon dioxide insufflation during the transperineal approach. In the transabdominal approach, adhesion of the small intestine and greater omentum to the abdominal wall was observed. Even after dissection of the intestinal and omental adhesion, it was difficult to recognize the wall of the remaining rectum because of the pelvic adhesion due to previous operations. Careful dissection of the rectum progressed laparoscopically. In the transperineal approach, the correct plane could be recognized and TME smoothly progressed. The influences of the inflammation of CD or chronic renal failure were none or very little to dissect the correct plane. The rectal dissection progressed along with the levator ani muscle, and the anococcygeal ligament was resected in the transperineal approach (Fig. 2a). Rendezvous of the abdominal space with the transperineal space was achieved on the dorsal side of the rectum. TME progressed from the posterior to lateral side of the rectum laparoscopically and transperineally. Preservation of the S4 nerve was confirmed in the transperineal approach. Finally, the anterior wall of the rectum was completely dissected after confirming the posterior wall of the prostate in the laparoscopic approach (Fig. 2b). The specimen was extracted from the perineal incision. Figure 2c shows the pelvic space after extraction of the specimen. Strong adhesion was observed in the pelvic floor. The operation was finished without conversion from laparoscopy to open surgery (Fig. 2d). The duration of the operation was 3 h 48 min, and the blood loss volume was 50 mL. No intraoperative complications occurred.

**Fig. 3:**
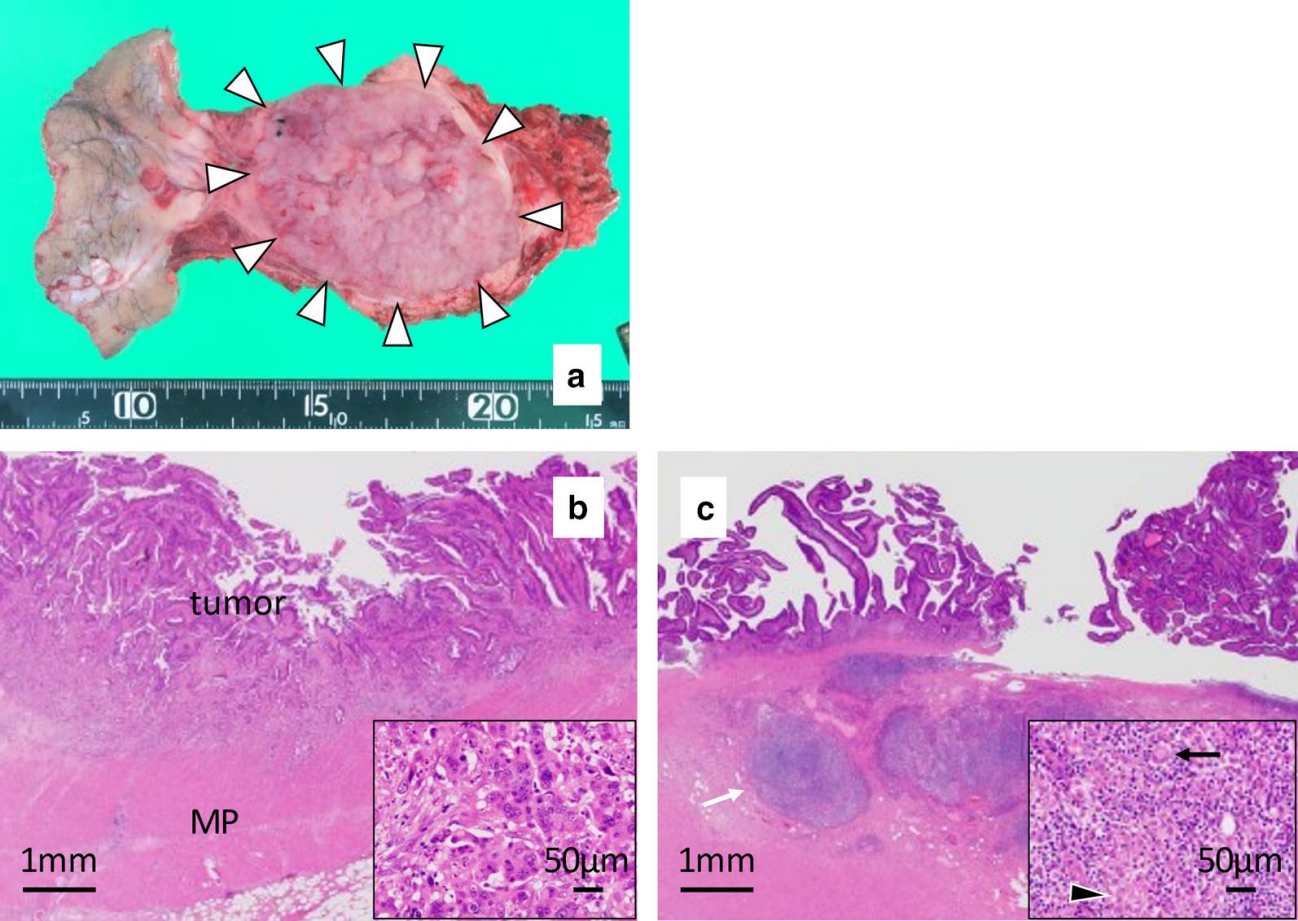
Pathological findings. **a** Resected specimen. White arrowheads show the tumor at the residual rectum. **b** Pathological examination of the rectal tumor with hematoxylin and eosin staining (× 40 and × 200). The depth of tumor invasion was pathologically diagnosed as T2. Columnar cells with hyperchromatic nuclei proliferating in tubular patterns were seen. Focal non-glandular irregular nests were also observed. The pathological diagnosis was well- to moderately differentiated adenocarcinoma with foci of a poorly differentiated component. MP: muscularis propria. **c** Pathological examination of the resected residual rectum with hematoxylin and eosin staining (× 40 and × 200). At low magnification, mononuclear cells (white arrow) were found to have migrated into the submucosal layer. At high magnification, noncaseous epithelioid cell granulomas (black arrowhead) and multinucleated giant cells (black arrow) were seen. These findings are typical pathological observations in patients with Crohn’s disease

On gross examination of the resected specimen, the tumor was a flat and villiform mass classified as type 5 (Fig. 3a). The size of the tumor was 80 × 67 mm. Histologically, the tumor was a well- to moderately differentiated adenocarcinoma with foci of a poorly differentiated component (Fig. 3b). The background contained characteristic findings of CD: chronic inflammation through the rectal wall and noncaseous epithelioid cell granulomas with multinucleated giant cells in the submucosa (Fig. 3c). Therefore, we diagnosed the tumor as colitic cancer associated with CD. The pathological stage was pT2N0M0, Stage I. The resection margin was negative.

During the postoperative course, the patient developed a fever due to an intra-abdominal infection 3 days after the operation, which might be due to a retrograde drainage tube infection estimated by CT, and it was treated with antibiotics. He was discharged 21 days after the operation. No recurrence was evident 3 months after the operation, and no adjuvant chemotherapy was performed.

## Discussion

We have herein presented a case of CD with rectal cancer at the residual rectum after multiple abdominal surgeries; the cancer was successfully treated with Ta-TME. Approximately 70 to 80% of patients with CD reportedly undergo surgical treatment [11, 12] and the rate of clinical anastomotic recurrence without drug therapy is around 20 to 25% per year [13]. Although the long-term impact of improvements in pharmaceutical and biological therapy on surgical outcomes is still unknown, it is considered that many patients with CD might undergo several surgical treatments. It is clear that the more frequently patients undergo surgical treatment, the more difficult transabdominal operations become because of the development of intra-abdominal adhesions. The patient in this report underwent three abdominal operations before rectal cancer surgery. The difficulty of the operation for the residual rectal cancer was easily predicted, and severe adhesion was in fact observed during the laparoscopic surgery.

Cancer at the defunctioning residual rectum or rectal stump is also reportedly problematic in patients with Crohn’s coloproctitis after total colectomy [3, 4, 14], although colorectal cancer rarely occurs in the entire cohort of patients with CD. In patients with CD, end ileostomy with a closed rectal stump is sometimes selected for treatment of Crohn’s colitis because perianal complications or impaired healing of a perianal wound may occur [15, 16]. Although fecal diversion may reduce the rectal inflammation, rectal cancer at the residual rectum can develop. Lutgens et al. [3] reviewed the literature of rectal stump cancer, including 29 patients with CD. They showed that one of the risk factors for rectal stump cancer was the duration of the disease. von Roon et al. [2] reported that the risk of developing colorectal cancer in patients younger than 30 years of age at the time of diagnosis of CD was 9.50 to 21.46 times higher than that in the general population. Whereas the risk of CD-associated rectal cancer was reportedly comparable with the risk of rectal cancer in the general population [2, 17], a multicenter investigation in France revealed that the incident ratio of rectal cancer in patients with CD who developed a perianal inflammatory lesion was 0.77 cases per 1000 patient-years and that the risk of rectal cancer in patients with CD who developed a perianal lesion was 5.11 times higher than that in patients without a perianal lesion [18]. These data indicate that a longer duration of extensive Crohn’s coloproctitis increases the risk of rectal cancer. We previously described a patient with CD who developed colitic cancer in the residual rectum following subtotal colectomy [19]. He had developed CD at 20 years of age and underwent subtotal colectomy at 37 years of age; the residual rectal cancer developed 8 years after subtotal colectomy. The risk of residual rectal cancer was deemed to be high in our patient because he had been diagnosed with CD at 16 years of age and had a history of perianal inflammation. Although his cancer was not derived from the scar of the anal fistula, pathological findings showed inflammation of the remnant rectum; this inflammation may have caused his rectal cancer.

Since Ta-TME was at first reported by Lacy and Adelsdorfer in 2011 [20], this procedure has become widespread throughout the world. It is difficult to achieve TME for mid- or low-rectal cancer because the accessibility from the abdominal cavity to the distal rectum is reduced by the forward angle of the low rectum [8]. In addition, obesity, a narrow pelvis, a bulky tumor, and pelvic irradiation make access to the low rectum and completion of TME more difficult [5, 6]. The transanal approach may provide clear visualization and detection of the correct dissection layer, facilitating complete TME [7]. The potential benefits of Ta-TME are expected to include higher-quality TME with a better CRM, less morbidity, a lower frequency of conversion, and more sphincter-saving resection [8]. Indeed, some of these benefits have been reported [6, 7, 21, 22]. Ta-TME is also considered to be useful in patients with a history of multiple abdominal surgeries. Narihiro et al. [9] reported a case of rectal cancer treated with Ta-TME in a patient who had undergone cholecystectomy, appendectomy, and Hartmann’s operation for sigmoid colon cancer. They concluded that they could overcome the difficulties of the transabdominal approach by Ta-TME; that is, the risks of bleeding and organ injury, longer duration of surgery, poor visual field due to intra-abdominal adhesion, poor surgical maneuverability, and loss of curability. In our case, although severe intra-abdominal adhesion was observed as predicted before the operation, Tp-TME facilitated detection of the correct plane of TME from the anal approach, reduced the duration of surgery by a two-team approach, avoided injury of organs adjacent to the rectum, and achieved negative pathological resection margin. The two-team approach also helped with the rectal dissection after rendezvous of the transabdominal and transperineal approaches. The appropriate dissection layer can sometimes be identified by applying tension to the tissue from both above and below. In addition, the advantages of Tp-TME also maximized in our case although the dissection between the rectum and the prostate was eventually performed laparoscopically. To dissect between the rectum and the prostate safely, the dorsal side of the prostate should be exposed from the lateral side. Although we finally dissected anterior side of the rectum laparoscopically, the transperineal approach played an important role to identify the correct layer between the rectum and the prostate.

Ta-TME is technically demanding and requires a proper anatomical perspective from the anal side. The data of the international registry of first Ta-TME cases revealed that misdirection of the correct layer occurred in 7.8% of cases and that injury to adjacent organs, including the urethra, occurred in 1.5% of cases [23]. Recently, data from the national registry of Norway showed a high rate of local recurrence in patients who had undergone Ta-TME for rectal cancer [24]. In this report, the 2-year local recurrence rate was > 10%, which was significantly higher than that associated with the laparoscopic approach. The authors speculated that gas pressure and surgical manipulation stressed the purse-string suture used to close the rectal lumen, resulting in spread of the cancer cells from the rectal lumen to the intra-abdominal cavity. In our department, a double purse-string suture is applied to tightly close the rectal rumen and prevent the spread of tumor cells. The international Ta-TME educational collaborative group provided recommendations for surgeons and centers as well as a training curriculum of Ta-TME [25]. Surgeons should carefully introduce this procedure to their own institute, determine the indications for Ta-TME, and train their own clinicians according to the expert guidance.

## Conclusions

We experienced a case of residual rectal cancer treated by combination of a laparoscopic and transperineal approach in a patient who had undergone several surgeries for Crohn’s enterocolitis. The development of rectal cancer might be a concern in patients with extensive Crohn’s coloproctitis, especially in those who were diagnosed with CD at < 30 years of age or who have had a long duration of CD. Increasingly more patients with CD are undergoing multiple abdominal operations as the prognosis of CD has improved, and Ta-TME or Tp-TME might be useful in patients with CD who develop rectal cancer after undergoing multiple abdominal surgeries.

## Data Availability

The data are not available for public access because of patient privacy concerns, but are available from the corresponding author on reasonable request.

## Abbreviations

CD: Crohn’s disease
CRM: Circumferential resection margin
Ta-TME: Transanal total mesorectal excision
TME: Total mesorectal excision
Tp-TME: Transperineal total mesorectal excision
